# Using Wearables and Machine Learning to Enable Personalized Lifestyle Recommendations to Improve Blood Pressure

**DOI:** 10.1109/JTEHM.2021.3098173

**Published:** 2021-07-19

**Authors:** Po-Han Chiang, Melissa Wong, Sujit Dey

**Affiliations:** 1 Mobile Systems Design LaboratoryDepartment of Electrical and Computer EngineeringUniversity of California at San Diego 8784 La Jolla CA 92092 USA; 2 Department of MedicineUniversity of California at San Diego 8784 La Jolla CA 92092 USA; 3 Primary Care UnitUC San Diego Health San Diego CA 92103 USA

**Keywords:** Blood pressure, hypertension, machine learning, personalized modeling, smart healthcare

## Abstract

*Background:* Blood pressure (BP) is an essential indicator for human health and is known to be greatly influenced by lifestyle factors, like activity and sleep factors. However, the degree of impact of each lifestyle factor on BP is unknown and may vary between individuals. Our goal is to investigate the relationships between BP and lifestyle factors and provide personalized and precise recommendations to improve BP, as opposed to the current practice of general lifestyle recommendations. *Method:* Our proposed system consists of automated data collection using home BP monitors and wearable activity trackers and feature engineering techniques to address time-series data and enhance interpretability. We propose Random Forest with Shapley-Value-based Feature Selection to offer personalized BP modeling and top lifestyle factor identification, and subsequent generation of precise recommendations based on the top factors. *Result:* In collaboration with UC San Diego Health and Altman Clinical and Translational Research Institute, we performed a clinical study, applying our system to 25 patients with elevated BP or stage I hypertension for three consecutive months. Our study results validate our system’s ability to provide accurate personalized BP models and identify the top features which can vary greatly between individuals. We also validate the effectiveness of personalized recommendations in a randomized controlled experiment. After receiving recommendations, the subjects in the experimental group decreased their BPs by 3.8 and 2.3 for systolic and diastolic BP, compared to the decrease of 0.3 and 0.9 for the subjects without recommendations. *Conclusion:* The study demonstrates the potential of using wearables and machine learning to develop personalized models and precise lifestyle recommendations to improve BP.

***Clinical and Translational Impact Statement–*** Our research demonstrates prospects for reducing BP through precise lifestyle changes, either effectuated through personalized interventions by clinicians, or patients following an interactive lifestyle coach with precise recommendations. (Category: Early/Pre-Clinical Research)

## I. Introduction

High blood pressure, or hypertension is one of the most prevalent chronic diseases in the world [1]. Stepwise management of hypertension begins with modifying lifestyle factors (e.g., activity, sleep) which, alone, can be effective in controlling BP [2]–[5]. What remains lacking in the literature is the individual effect of these lifestyle factors on BP. Traditionally, these relationships have been investigated through large- scale Randomized Controlled Trials (RCTs). However, the aggregate insights derived from RCTs are not necessarily tailored for individuals. That is, the impact of specific lifestyle factors on BP may differ across individuals due to an individual’s unique genomic makeup. Secondly, the data in the RCTs are usually collected in healthcare settings or self-reported fashions. It is well-established that BP measurements obtained in healthcare settings are often unreliable [6], while self-reported data often falls short of accuracy and granularity.

In contrast, wearable activity trackers, or wearables, such as Apple Watch, Fitbit and Samsung Galaxy Watch, collect a great amount of lifestyle data in high granularity and continuity. As a result, a personalized model for BP and lifestyle factors can be built for each individual based on his/her data. To date, the potential of using wearables’ data for BP management has not been fully investigated due to the complex dependency between BP and lifestyle factors. In this study, we propose to use machine learning (ML) techniques to elucidate the complex relationships between BP and lifestyle factors at the level of the individual. Based on the continuous data collected from wearables of users, we aim to 1) build a predictive model of BP for individuals, which will give users a quick and reliable way to understand their health condition, and 2) utilize the above model to provide personalized and precise insight to users, as opposed to general lifestyle recommendations.

In our preliminary work [7], we used Fitbit Charge HR and Omron Evolv to collect lifestyle and BP data, respectively, of 8 volunteers. We then trained a Random Forest (RF) model [8] to predict the 24-hour-ahead BP for each volunteer using lifestyle data. We proposed a stable and consistent feature selection technique, namely Random Forest with Feature Selection (RFFS), to enhance the prediction accuracy of RF. Moreover, we used the relative importance of the features generated by RFFS to identify the most important lifestyle factors for his/her BP. The most important lifestyle factors were shared with selected subjects. We observed that the above subjects changed their lifestyle factors according to the shared information and their BP decreased from its previous level. In [9], we proposed an online ML technique to prioritize training samples based on the performance of prediction. The proposed technique addressed the challenge of concept drifts and anomaly points due to sequential data collection.

However, there were three main limitations to be addressed: 1.) The dataset consisted of a series of BP and lifestyle factors data with mixed sampling frequency. Extra feature engineering and modeling for time series were necessary to fully utilize the potential of temporal dependency. 2) The selection of the feature in RFFS was based on how each feature improved the prediction accuracy of BP; however, it did not imply how each feature is affecting BP. 3.) The recommendation was only given to two subjects, and the duration of observation after the recommendation was only one week. The lack of a control group and short observation time made it challenging to reach a significant conclusion.

To tackle the above challenges, we extract new features from raw data collected by wearables and BP monitors. We aggregated the raw lifestyle data, which was mostly recorded every minute, into a summary of 1-hour, 24-hours, 48-hours and 72-hours before each BP reading and extracted features with the above non-overlapping and contiguous time windows. The improved granularity and representation of features extracted from wearables are not only improving the accuracy of BP prediction but also comprehendible for patients and physicians. Secondly, to capture the periodicity and the trend of previous BPs, we create new features based on Autoregressive Integrated Moving Average (ARIMA) model [10] to better represent the BP time series. To deal with unevenly spaced BP readings, we propose to transform the original BP time series into an evenly spaced time series by resampling and interpolation. To explore the best feature selection method, we evaluate multiple popular methods, and we choose Shapley value [11] based on its prediction performance and interpretability. Shapley value is a model-agnostic feature interpretation method derived from Game Theory. Given a set of feature values and a trained ML model, Shapley value can indicate how each feature contributes to the actual BP prediction from the mean prediction. We propose a feature selection method, namely RF with Shapley-Value-based Feature Selection (RFSV), which uses feature importance based on Shapley value to remove redundant and irrelevant features. Moreover, we use the top features selected by RFSV to provide the precise insight that may affect an individual’s BP.

To evaluate the effectiveness of the proposed techniques, we conducted a randomized controlled experiment with patients who have Elevated BP or Stage I hypertension and were not taking any antihypertensive medications. We collected BP and wearable data and trained the BP prediction model for each subject. Subjects were randomized to either receive personalized lifestyle recommendations based on their data (experimental group) or not receive lifestyle recommendations (control group). We compared and discussed the change of BP levels across the study period for both groups.

The rest of the paper is organized as follows. In Section II, we will investigate the related work of BP prediction technique and BP studies using lifestyle intervention. In Section III, we present the overall architecture of the BP prediction and recommendation system with the proposed RFSV. We then detail data collection and representation, ARIMA time series feature extraction and RFSV. In Section IV, the prediction performance of the proposed method is compared with other ML methods. Moreover, we will discuss the effectiveness of personalized lifestyle factors recommendations suggested by the proposed system. Finally, we conclude the paper in Section V.

## II. Related Work

The authors in [13] predicted BP using demographic and contextual data (e.g., age, weight and smoking habit) with an artificial neural network (ANN). However, the prediction was based on a single BP measurement and did not consider the dynamics of BP. In [14]–[18], PPG signals were used to predict short-term BP with ensemble trees models [14]–[16] and neural-network-based models [17], [18]. However, PPG-based prediction is only applicable for a very short time horizon (~10 minutes), while our technique aims to predict BP in a longer time horizon, to provide actionable information to users. In [19], the 24-hour time series of BP and heart rate were trained with Extreme Learning Machine (ELM) to provide hourly BP prediction. However, the length of collected data in [19] was only a single day, and the prediction performance was not compared with other ML methods. The authors in [20] proposed to predict BP using Long Short-Term Memory (LSTM) models [47] with additional contextual data (e.g., age, BMI and BP medication) layer. The data in [20] was averaged every month, so the temporal relationship of data was not fully utilized due to lower temporal resolution and information loss in the averaging process. All the above studies did not use physical activity and sleep data, which were the most relevant lifestyle data related to BP that can be collected by current technology. During physical activity, heart rate and stroke volume increase to meet the metabolic requirements of the muscles, which result in expansion of arteries and force exerted against the artery changes, which is translated into BP [21]. Although BP normally increases during physical activity, the inverse relationship between physical activity and BP has been shown in numerous observational studies and can be explained by the reduction of arterial stiffness through exercise [22]. Secondly, inadequate sleep, including issues of quantity and efficiency, also has a significant negative impact on BP, possibly by higher hypothalamic-pituitary-adrenal axis activation [23]. Besides activity and sleep factors, it has been known that dietary factors, like sodium intake, may also affect BP [2], [24]. Traditional methods assess food (nutrition) intake with self-report measures, such as food frequency questionnaires (FFQs) and photo-assisted dietary assessments [25]. However, the accuracy of dietary intake assessment remains a challenge. Moreover, no widely adopted technology can assess dietary intake automatically and accurately [25]. Therefore, we focus on only physical activity and sleep factors in our study. Based on heart rate and steps collected by wearables, the authors in [26] trained bidirectional LSTM models to diagnose various chronic diseases, including hypertension. However, the proposed methods focused on the diagnosis of hypertension and did not provide a numerical prediction of BP.

In addition to BP prediction, the other critical insight from BP analysis is how lifestyle factors such as physical activity and sleep affect an individual’s BP. Although the effectiveness of lifestyle interventions on BP management has been proven in many studies [2]–[5], the insight on an individual level is absent. Long-term BP and the result of exercise treadmill stress tests were used for BP factor analysis in [27]. The authors compared different interpretable ML techniques and concluded that those techniques could derive different insights on the model behavior. In [28], a mobile app was designed to deliver behavioral recommendations on diet and exercise to manage hypertension. The authors in [28] collected biometric, demographic and engagement data from a mobile app, and they proposed ML models to predict participant completion of the intervention. The BP factors collected by the above studies were either from electronic health records or self-reported methods, so the accuracy and granularity of lifestyle factors were limited. In contrast, our method uses wearables to collect lifestyle data, which enhances the quality and granularity of the data. Therefore, our model can pinpoint the lifestyle factors responsible for an individual’s BP. Moreover, the conclusions of previous studies are only drawn from ML models without validating the effectiveness of the recommendations. In our study, we provide recommendations based on Shapley Value and conduct a randomized experiment to validate the effectiveness of recommendations.

## III. Method

In this section, we will first introduce the clinical study and data collection process. We will give an overview of the BP prediction and lifestyle recommendation system and discuss each step in more detail.

### A. Clinical Study Cohort and System Architecture

**FIGURE 1.**
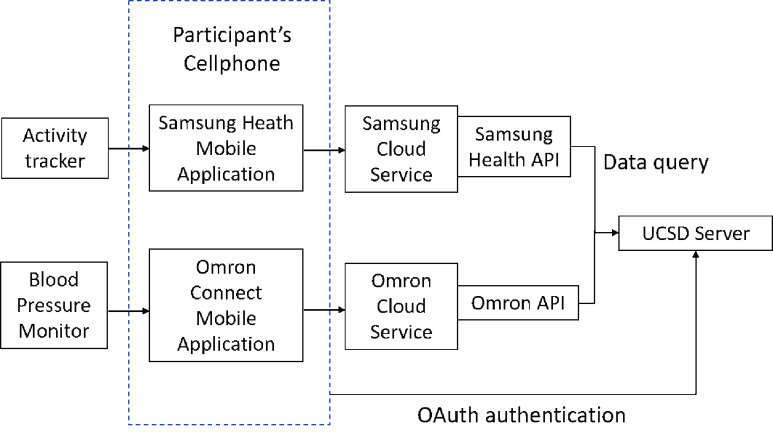
Block diagram of data storage and access.

Our clinical study (protocol #181405) was reviewed and approved by UC San Diego Human Research Protections Program, which operates Institutional Review Boards (IRBs) at UC San Diego. The study was in collaboration with UC San Diego Health, with patient enrollment, onboarding and management conducted by the Altman Clinical & Translational Research Institute at UC San Diego. Patients were screened for recruitment with UC San Diego Health System’s electronic health record. The selection criteria included subjects who were pre-hypertensive or with Stage I hypertension (SBP between 120-140/DBP under 90 per ACC/AHA 2017 guidelines [12]) and who were not taking any antihypertensive medications. Subjects who had consented were provided a Samsung Galaxy Watch and an Omron Evolv wireless BP monitor to collect their lifestyle factors and BP data for 90 days. Of the 36 consented subjects, data of 11 subjects were excluded since they withdrew from the study or failed to collect data for at least half the study duration (45 days) in the study period. The characteristics of the included cohort are shown in Table 1. Data was collected remotely through the application programming interfaces (APIs) provided by Samsung and Omron, as shown in Fig. 1. The primary metrics used to measure BP are systolic and diastolic blood pressure (SBP and DBP), which are defined as the maximum and minimum BP, respectively, during a pulse.

**TABLE 1:** Cohort Statistics (n = 25)

Age (yrs +/-SD)	50.2+/- 14.3
Male	16
Female	9
SBP (Mean +/- SD)	126.2+/- 8.3
DBP (Mean +/- SD)	78.5+/- 6.3
Average Resting Heart rate	69.6+/- 10.1
Peak Heart rate	92.9+/- 12.3

**FIGURE 2.**
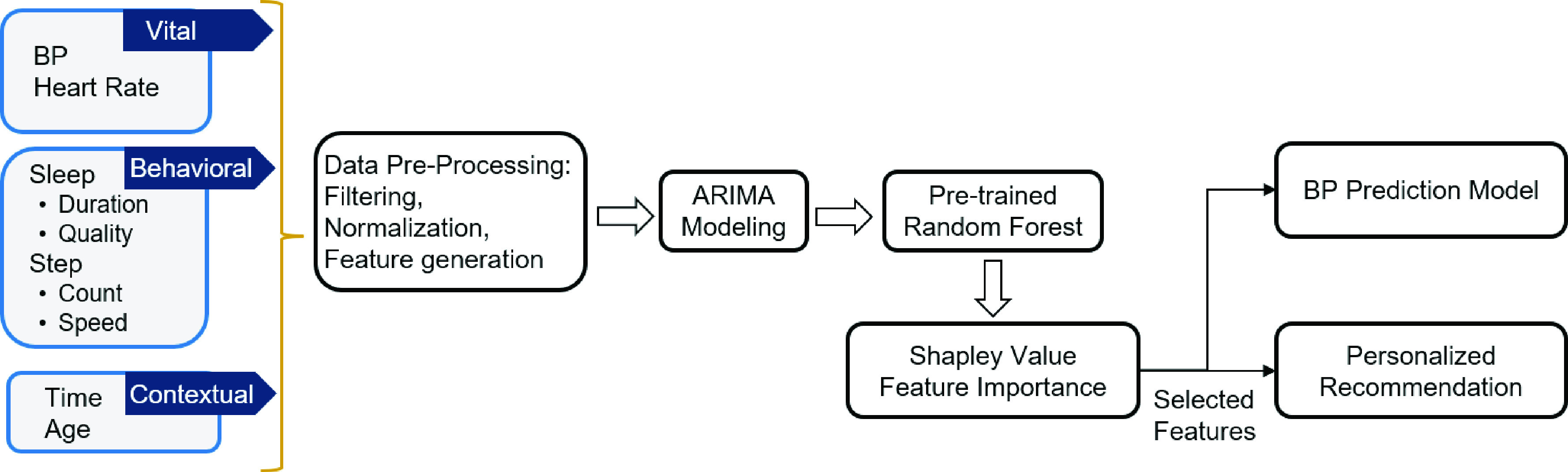
System architecture of the proposed method.

The objectives of our proposed system, shown in Fig. 2, are prediction of BP for an individual, identification of the most important features that impact the individual’s BP trend and providing personalized and precise recommendations on lifestyle factors that will positively impact his/her BP trend. To achieve the objectives, we train a ML model to predict the current BP level using one’s historical BP readings as well as activity, sleep and heart rate data collected from the Galaxy Watch. The raw data are then filtered, extracted and imputed as features. To better capture temporal information in BP time series, we extract time-series features of BP using ARIMA, as discussed in Sec. III-C. The feature selection based on a pre-trained RF model and Shapley value is performed to remove redundant and/or irrelevant features in BP prediction. In addition to building a predictive model of BP, we will provide personalized lifestyle recommendations to our subjects by pointing out the most important factors affecting their BP based on Shapley value.

### B. Data Characteristics and Features Extraction

The Galaxy Watch provides heart rate (HR), number of steps, walking/running speed, floors climbed, sleep duration and sleep stages of the user. Also, we discretize the activity data into different levels of active time (sedentary, lightly active, very active) based on subjects’ steps and heart rate every minute. Maximum HR (${HR}^{max}$) of each subject is calculated as [29]:

\begin{equation*}220-age.\tag{1}\end{equation*}

Three HR zones (zone 1, 2, and 3) are defined as [27]:

\begin{equation*} Z\ast {HR}^{max},\quad Z\in \left [{.5,.7,1 }\right].\tag{2}\end{equation*}

We define three active levels as follows: sedentary (steps < 10 or HR in zone 1), lightly active (steps ≥10 and HR is in zone 2), and very active (steps ≥10 and HR in zone 3). Sleep data includes sleep duration, bedtime, wake-up time and sleep stages. Bedtime and wake-up time represent the time subjects go to sleep and wake up, respectively. Sleep stages include light sleep, REM sleep and deep sleep. We also define the average heart rate during sleep as slpHR.

**FIGURE 3.**
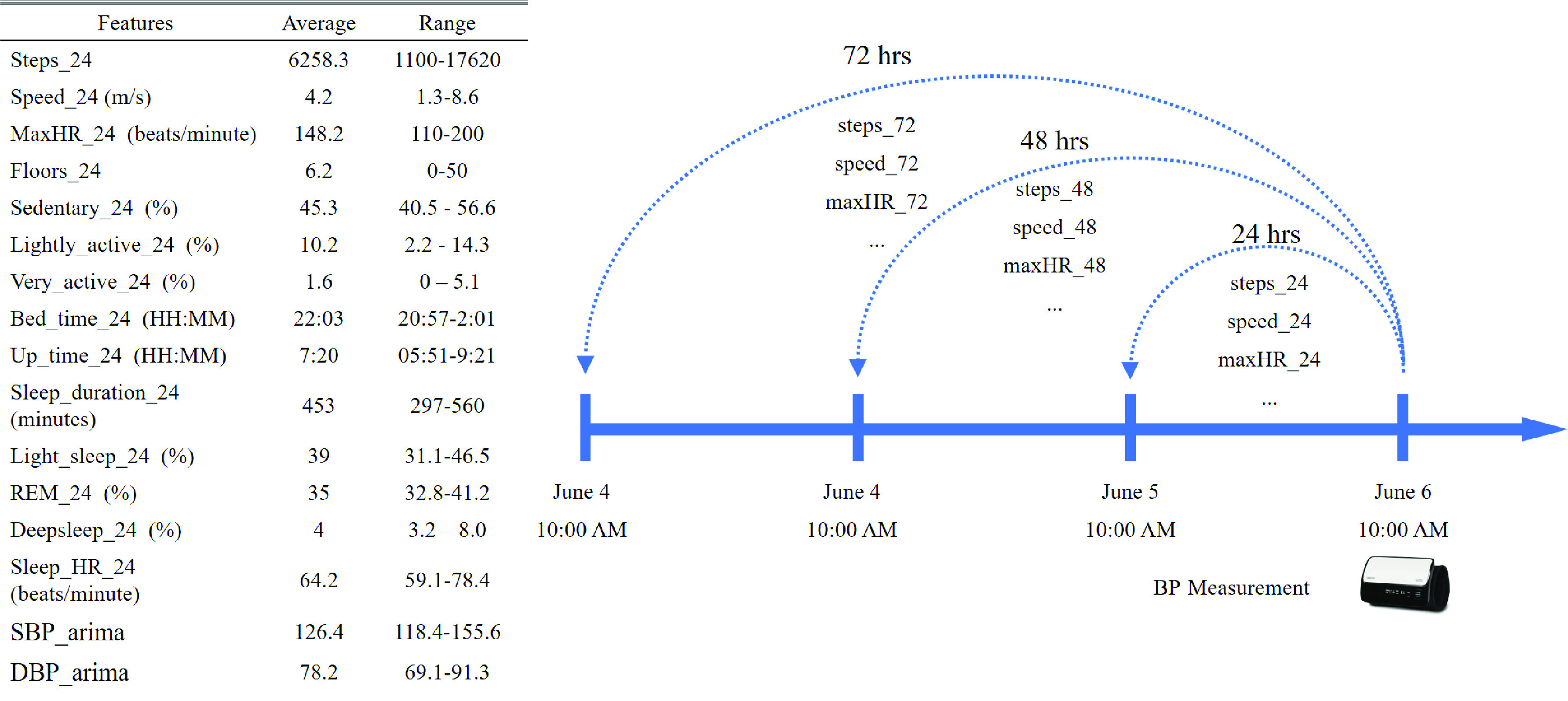
Left: Statistics of representative features. Right: Illustration of feature extraction.

Data from the Galaxy Watch is mostly recorded every minute while BP is measured by subjects twice per day, so the data consists of time series with different frequencies. Moreover, although the guideline for BP measurement in this study is to measure in the morning (8-10 am) and at night (7-9 pm), there are missing values, time deviations (e.g., measurement in the afternoon) and redundant values (e.g., two-morning measurements at 7 am and 9 am, respectively). Thirdly, most of the lifestyle factors such as sleep and activity, have a daily cycle. Based on the above observations, we extracted the lifestyle factors data as a summary of 24-hours, 48-hours and 72-hours before each BP reading and extracted features using the above non-overlapping and contiguous time windows. For example, for each pair of (SBP, DBP), the feature “steps_24” was defined as the total number of steps in the previous 24 hours before the measured BP and “step_48” was the average of the total daily steps in the previous 48 hours. Note that instead of summation, HR and walking/running speed were averaged over the previous 24/48/72 hours and MaxHR is the maximum HR over the previous 24/48/72 hours. Finally, “measure_time” denotes the time in a day when BP was measured. The statistics of the representative features over the previous 24 hours and the method of feature extraction are shown in Fig. 3. The original and derived features are summarized in Table 2.

**TABLE 2:** Features and Target Variables

Target Variables	Original Features	Derived Features
SBP, DBP	heart_rate_24, maxHR_24, steps_24, speed_24, floors_24, bed_time_24, up_time_24, sleep_duration_24 (sleep_24), light_sleep_24 (Lsleep_24), REM_24, deepsleep_24 (Dsleep_24), heart_rate_48, maxHR_48, steps_48, speed_48, floors_48, bed_time_48, up_time_48, sleep_duration48 (sleep_48), light_sleep_48 (Lsleep_48), REM48, deep_sleep48 (Dsleep_48), heart_rate_72, maxHR_72, steps_72, speed_72, floors_72, bed_time_72, up_time_72, sleepduration72 (sleep_72), light_sleep_72 (Lsleep_72), REM72, deep_sleep_72 (Dsleep_24), measure_time	SBP_arima, DBP_arima, sedentary_24, lightly_active_24, very_active_24, slpHR_24, sedentary_48, lightly_active_48, very_active_48, slpHR_48, sedentary_72, lightly_active_72, very_active_72, slpHR_72,

### C. ARIMA Feature Extraction from BP Time Series

Time series prediction problems include a set of time-ordered observations $s_{j}=\left ({X_{j},y_{j} }\right),j=1,2\ldots J$ where $X_{j}$ are the values of features $X$ and $y_{j}$ is the value of target $y$ observed at time $j$, and the task is defined as predicting the future values of $y_{u}$ for time $u>j$ given $s_{1}{,s}_{2}\ldots,s_{j}$. In addition to using $X$ as features, time-series features can be extracted from $y_{1}{,y}_{2}\ldots,y_{j}$ to capture the temporal relationship of $y$. In this paper, we use ARIMA [10] to capture the temporal pattern of BP series.

Three parameters $(p,d,q)$ are used to construct the ARIMA model, and $(p,d,q)$ stands for the order of the autoregressive model, the order of differencing, and the order of the moving average model, respectively, and the prediction $y_{j}$ can be expressed by:

\begin{align*}&\hspace {-.5pc} {(1-S)}^{d}y_{j}=\delta +\alpha _{1}y_{j-1}+\alpha _{2}y_{j-2}+\ldots +\alpha _{p}y_{j-p}+\varepsilon _{1} \\&\qquad\qquad\qquad\qquad-\beta _{1}\varepsilon _{j-1}-\beta _{2}\varepsilon _{j-2}-\ldots -\beta _{q}\varepsilon _{j-q}\tag{3}\end{align*}

where $S$ stands for the backward shift operator for $S\left ({y_{j} }\right)=y_{j-1}$, $\delta $ is the constant, $\alpha _{1},\alpha _{2,}\ldots,\alpha _{p}$ are the autoregressive parameters, $\varepsilon _{j}$ is the random error at time $t$ and $\varepsilon _{j}\sim N(0,\sigma ^{2})$, and $\beta _{1},\beta _{2},\ldots,\beta _{q}$ are the moving average parameters. To cope with seasonality, the authors in [30] proposed Seasonal ARIMA (SARIMA). In SARIMA, additional seasonal AR and MA terms are used for prediction using values at times with lags that are multiples of pre-defined periods $T$ (the span of the seasonality). In this paper, we set $T=1$ days. To determine these three parameters $(p,d,q)$, we run an exhaustive search to determine the best ARIMA model for each subjects’ BPs and the corresponding set of optimal parameters. After the model is developed, one-step forecasts from the ARIMA model are defined as additional features, namely SBP_arima and DBP_arima.

As described in the previous section, the BP series is not evenly spaced due to manual measurements. For example, a subject may measure his/her BP at 7 am, 3 pm and 9 pm on one day while measuring his/her BP only at 6 pm on another day. However, ARIMA can only model evenly spaced time series. To address this issue, we transform the BP data into evenly spaced observations by resampling and linearly interpolating the closest two BP readings before and after each resample point. Note that the resampled BP series is not the actual BP measurement and is used only to generate ARIMA features.

### D. Predictive Modeling Using Random Forest (RF)

To select the best ML methods for our task, we evaluate popular machine learning techniques, including Random Forest, Support Vector Machine [45], Gradient Boosting Decision Trees [46], LSTM [47], and ARIMA [10]. Although neural network-based approaches outperform in unstructured data like image and language, tree-based ensemble ML models constantly have the best performance in structured data where data is essentially in tabular form [31]. Moreover, neural networks are highly prone to overfitting when the underlying data sizes are small and no domain-specific insight can be used to design the architecture of the underlying neural network [32]. In this study, the number of BP samples for each subject is less than 180 (subjects are requested to measure their BP twice per day for 90 days) and the data is structured for interpretation purposes, which is best suited for tree-based ensemble ML models. Among the ML models, we find that RF gives the best performance through the evaluation in Sec. IV-B (Table 3). Therefore, RF is used to model BP and lifestyle factors in this study.

**TABLE 3:** Prediction Error of RFSV and Related Models

	SBP				DBP			
	MAE	RMSE	MAPE (%)	R^2^	MAE	RMSE	MAPE (%)	R^2^
SimpleMean	6.87	10.32	5.46	0.29	4.70	7.86	5.99	0.27
SVM	6.15	9.67	4.87	0.46	4.26	7.21	5.40	0.44
GBDT	6.03	9.64	4.71	0.46	4.11	7.01	5.22	0.45
LSTM	8.21	13.10	6.64	0.21	6.01	10.23	7.54	0.19
ARIMA	5.94	9.98	4.70	0.41	4.05	6.68	5.24	0.47
RFSV	5.34	8.24	4.19	0.51	3.80	6.05	4.83	0.52

RF is an ensemble predictor of several decision tree predictors. We will first introduce the decision tree and its application in ML tasks, Classification and Regression Tree (CART) model. CART [33] is a non-parametric method used to build decision tree predictors in ML problems. CART arranges a sequence of questions (decision rules) based on input features into a tree-lie structure. A decision tree consists of two types of nodes: 1) internal nodes, which split the samples into two sub-trees or leaf nodes based on decision rules. Each internal node is labeled with a single input feature and a corresponding split threshold of that feature. 2) leaf nodes, where no more split is performed. In regression tasks, the target variable is continuous, so the prediction of the target variable is the average of all training samples at that node. In the training phase, the topmost internal node (root node) contains all training samples. At each internal node, the feature and its split threshold are selected to minimize the mean squared error of the prediction. In the prediction phase, the new sample moves down from the root node to one of the leaf nodes according to the splitting criteria along its path. The predicted value is then the average training sample at that leaf node.

RF is an enhanced approach by aggregating a collection of decision trees to reduce overfitting of the data and the resulting high variance of the prediction [8]. Compared with CARTs, RF introduces two major enhancements: bootstrap aggregation (bagging) and feature bagging. RF produces bootstrap datasets that are randomly and independently drawn with replacement from the training dataset. Each bootstrap dataset with the same size as the original training set is used to train a decision tree. Bootstrap aggregation in RF averages the prediction of decision trees trained with bootstrap samples, which greatly reduces the variance of prediction from a single decision tree. Moreover, since individual trees generated in the bagging process are identically distributed, the expected prediction of RF is the same as the expected prediction of individual trees. As a result, RF has a lower variance than individual trees, while its bias remains the same [34]. In addition to bootstrap aggregation, RF further reduces the correlation between its member decision trees by introducing feature bagging, which randomly selects a subset of features when constructing each tree.

### E. Feature Importance with Shapley Value

Although RF performs well on classification and regression tasks, high-dimensional data will degrade the performance, especially when the number of samples is relatively small. There may be redundant features, which provide no more information than the currently selected features, or irrelevant features, which may introduce noise instead of any useful information.

Feature selection techniques improve the prediction accuracy and reliability by removing irrelevant or redundant features across the datasets. In our study, the candidate feature selection method should not only improve the prediction performance but also measure the relevance between BP and the features. With the relevant information, the most relevant (important) feature can be used for personalized and precise recommendations. Based on the above objectives, we choose four representative feature selection methods, namely, Pearson Correlation-based Feature Selection [35], Information Gain-based Feature Selection [36], Random Forest Feature Importance (mean decrease impurity) [37], and Shapley Value Feature Importance [38], [39]. All four methods provide a numerical importance or relevance measure for each feature, which can be used to select the features for ML tasks and provide recommendations based on the importance score. Based on the empirical evaluation of prediction accuracy, which is detailed in Sec. IV, we select Shapley Value Feature Importance to select the features.

Shapley value, derived from Game Theory, assumes that each feature in a data sample is a ‘player’ in a game, and the prediction is the payout [11]. The Shapley value aims to distribute the payout among the features based on their contribution. To calculate feature importance for each feature $x_{k},k=1,2\ldots K$, based on Shapley value, the model is evaluated over all possible feature value combinations with and without $x_{k}$. The Shapley value is calculated by [40]:

\begin{equation*} \phi _{k}=\sum \nolimits _{S\subseteq X\mathrm {\backslash \{}x_{k}\}} \frac {\left |{ S }\right |\mathrm {!}\left ({p-\left |{ S }\right |-1 }\right)\mathrm {!}}{p\mathrm {!}} (f(S\cup x_{k}\mathrm {)-}f(S\mathrm {)) }\tag{4}\end{equation*}

where $S$ represents all possible feature sets $S\subseteq X\backslash \{x_{k}\}$ and $X$ is the set of all features. $p$ is the number of the features in $X$ and $\left |{ S }\right |$ is the number of features in $S$. $f(S\cup \{x_{k}\})-f(S)$ is calculated by the marginalized prediction using the model trained with feature set $S\cup \{x_{k}\}$ minus the prediction using model trained with feature $S$.

The complexity to compute the exact form of $\phi _{k}$ is prohibitively high since the number of possible sets $S$ in (4) is $2^{n}$ where $n$ is the number of features. In [41], the authors propose Tree Shapley Additive exPlanation (SHAP) algorithm to approximate Shapley value in polynomial time for tree-based ML models. This algorithm has been used in this work to calculate the feature importance. By averaging the absolute value of all Shapley values across all training samples, we can get the average contribution of a feature to the prediction of our pre-trained model. We define the feature importance vector for the $j^{th}$ sample as $I_{Xy}(j) = [\phi _{1},\phi _{2}\ldots,\phi _{k}]$ where $j =1,2\ldots J,k=1,2\ldots K$. The average feature importance can then be calculated by

\begin{equation*} \bar {I}_{Xy}=\frac {\sum \nolimits _{j=1}^{J} {\left |{ I_{Xy}(j) }\right |} }{J}.\tag{5}\end{equation*}

### F. RF with Shapley-Value Based Feature Selection (RFSV) and Personalized Recommendations

**FIGURE 4.**
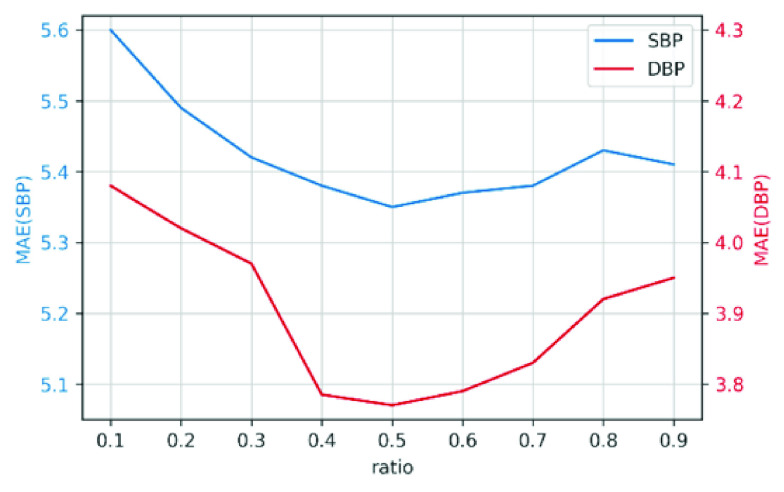
Prediction error with different ratios of selected features.

To select the best features for BP prediction, we first train a RF model using all features of training samples and calculate the feature importance $\bar {I}_{Xy}$ for all features. Based on $\bar {I}_{Xy}$, we select a subset of features with higher feature importance. To decide the selection ratio of total features, we compare the performance between different ratios. Fig. 4 shows the BP prediction performance, measured by mean absolute error (MAE) of the final RF models trained with features under different selection ratios. We can observe that the ratio of 0.5 performs the best in terms of MAE. Based on the empirical results, we select 0.5 as the ratio of feature selection. The resulting BP prediction model is the RF model re-trained with only the selected features based on $\bar {I}_{Xy}$.

In addition to prediction performance, Shapley value suggests how each feature contributes to the deviation of BP prediction from the average BP prediction among the dataset. Therefore, we select the top three lifestyle factors with the highestShapley importance for each person as his/her personalized and precise recommendation. Note that in the recommendation, we exclude measure_time, heart rate and BP time-series features derived in Sec. III-B, even if they are selected as the top factors. The rationale is that those factors are not actionable for subjects although they might contribute to BP prediction.

## IV. Results and Discussion

In this section, we will first discuss the experiment settings. We will present the results obtained by using the proposed RFSV and compare the results with existing ML models. Secondly, we will validate the effectiveness of personalized and precise recommendations of lifestyle factors generated by our BP model using RFSV.

### A. Experiment Setting

Of the 25 subjects, we sorted out the 13 subjects to train and evaluate the BP-lifestyle model based on the quality, length, and availability of their data. Each person’s model is trained with only his/her data. The other 12 subjects had sufficient BP data but less than 45 effective days of continuous lifestyle data. However, their BP data was included in Sec. IV-C as in the control group to evaluate the effectiveness of personalized recommendation. We implement and evaluate our proposed methods in the Python environment. We also use the Tree SHAP [41], Scikit-learn library [42], Keras [43] and Auto. Arima [44] to implement RFSV and other ML models. MAE, root mean square error (RMSE), mean absolute percentage error (MAPE) and Coefficient of determination ($R^{2}$) are calculated and used as our evaluation metrics. Their definitions are as follows:

\begin{align*} MAE=&\frac {\sum \nolimits _{i=1}^{n} \left |{ \widehat {BP}^{i}-{BP}^{i} }\right | }{n} \tag{6}\\ RMSE=&\sqrt {\frac {\sum \nolimits _{i=1}^{n} \left ({\widehat {BP}^{i}-{BP}^{i} }\right)^{2}}{n}} \tag{7}\\ MAPE=&\frac {nMAE}{\sum \nolimits _{i=1}^{n} \left |{ {BP}^{i} }\right | }\times 100\% \tag{8}\\ R^{2}=&1-\frac {\sum \nolimits _{i=1}^{n} \left ({\widehat {BP}^{i}-\widehat {BP}^{i} }\right)^{2}}{\sum \nolimits _{i=1}^{n} \left ({{BP}^{i}-\widehat {BP}^{i} }\right)^{2} }\tag{9}\end{align*}

where $\widehat {BP}^{i}$ is the $i$th prediction of BP made by trained models and ${BP}^{i}$ is the actual value of the $i$th BP.

We use 5-fold cross-validation to randomly split our dataset into training (80%), and test (20%) sets five times and average the prediction results. To show the effectiveness of RFSV, we compare the predictive performance with several representative ML algorithms referenced earlier in Sec. II, including Support Vector Machine (SVM), Gradient Boosting Decision Trees (GBDT), Long Short-Term Memory (LSTM), and ARIMA. We also compare our performance against a regressor (termed as SimpleMean), which always predicts the mean of the training data. The rationale is that the prediction error may largely depend on the underlying BP fluctuation of the subject. By comparing SimpleMean and other ML algorithms, we can exclude the dependency of the underlying fluctuation. In ARIMA, we take SBP_arima and DBP_arima, the ARIMA forecasts introduced in Sec. III-C. For setting details of other models, we set the number of trees to 500 for all RF models. We set the maximum ratio of total features used in each tree as 0.33 and the minimum number of samples to split as 2. For SVM, the RBF kernel is used, and the best $\gamma $ and $C$ are selected using cross-validation. For GBDT, the number of trees as 500 and the learning rate as 0.05. LSTM was trained using 0.001 and 20 as the learning rate and batch size with Adam optimizer [48]. The total depth of the fully connected layers in LSTM models was set to 4 and the maximum neurons in each layer to 50. We also use early stopping and dropout layers with a dropout rate of 0.2 to avoid overfitting.

### B. BP Prediction Using RFSV

The MAE and RMSE of BP prediction of the proposed method and other methods are summarized in Table 3. Note that the values in Table 3 are the average MAE and RMSE over all the users. As shown in Table 3, most of the methods outperform SimpleMean, which suggests the prediction power of lifestyle factors. The possible reason why LSTM performs the worst of all methods is LSTM may overfit the small training dataset (~ 180 samples for each user). GBDT and SVM perform similarly while GBDT has a slightly better prediction error. ARIMA is the second-best method based on MAE for SBP and MAE and RMSE for DBP. The possible reason is the temporal dependency in historical BP contains enough information, that with proper modeling, it outperforms ML models only based on lifestyle factors. However, worse RMSE for SBP using ARIMA may suggest overfitting to the SBP series. Among all methods, our proposed RFSV model achieves the lowest prediction error in terms of MAE, MAPE and RMSE. Our proposed RFSV performs better than ARIMA by 10.1% and 6.2% in terms of MAE for SBP and DBP; 10.9% and 7.5% in terms of MAPE for SBP and DBP; 14.4% and 10.4% in terms of RMSE for SBP and DBP, respectively (RMSE of SBP is compared with GBDT). In terms of $R^{2}$, RFSV achieve 0.51 and 0.52 for SBP and DBP, which means the most proportion of the variance is explained by RFSV compared to other methods.

We carry out a Paired Student’s t-test [49] separately for each subject to assess the statistical significance of the difference in estimation errors between our method RFSV and two methods, ARIMA and GBDT, which achieve the closest performance to our method shown in Table 4. The null hypothesis of the Paired Student’s t-test is that there is no difference between the performance of two ML models. We then calculate the p-value using the method in [49] for each subject and compare it with a significance level $\alpha $, the probability of rejecting the null hypothesis given that it is true ($\alpha =0.05$ is used in most studies). If the p-value is smaller than $\alpha $, the null hypothesis is rejected. Therefore, the results statistically provide convincing evidence that two ML models perform differently. For 16 out of the 25 subjects, the performance difference between RFSV and ARIMA is statistically significant at the level $\alpha =0.05$ for both SBP and DBP. Similarly, for 20 out of the 25 subjects, the performance difference between RFSV and GBDT show statistical significance at the level $\alpha =0.05$ for both SBP and DBP.

**TABLE 4:** MAE in Different Prediction Horizons Using RF, RF-ARIMA and RFSV MAE (SBP/DBP)

	Current	12hr	24hr	48hr
RF	6.15/4.26	6.89/5.16	6.45/4.83	6.79/5.01
RF-ARIMA	5.79/3.95	6.61/4.82	6.21/4.48	6.51/4.60
RFSV	5.34/3.80	6.32/4.5	6.01/4.21	6.25/4.38

Besides the prediction of current BP, we will discuss the effect of applying ARIMA prediction of BP and Shapley-based feature selection for different prediction time horizons. The BP predictions of current BP (the MAEs in Table 3), 12 hours, 24 hours and 48 hours ahead are summarized in Table 4, comparing our proposed RFSV with: 1) RF, which does not include SBP_arima and DBP_arima and feature selection, and 2) RF-ARIMA, which includes SBP_arima and DBP_arima but without feature selection. As shown in Table 4, we can make the following observations: 1) RFSV consistently gives the best BP prediction, which shows the effectiveness of ARIMA feature extraction and Shapley-based feature selection. 2) For each method, the MAE worsens as the prediction horizon expands, except for 12-hours ahead prediction, which is the worst performer. The result indicates that the accuracy of the prediction based on lifestyle factors and historical BP decreases with time. The worst performance for 12-hours ahead prediction suggests that the proposed technique may work better when the prediction horizons are multiples of 24 hours.

Finally, we compare the average MAE of RFSV (which uses Shapley value for feature selection) with three other feature selection methods introduced in Sec. III-E, namely Pearson Correlation-based Feature Selection (PCFS), Information Gain-based Feature Selection (IGFS), and Random Forest Feature Importance (RFFI). As shown in Table 5, all feature selection methods result in lower MAE than the prediction without feature selection. While RFSV and RFFI perform significantly better than PCFS and IGFS, RFSV has the lowest MAE. We also carry out a Paired Student’s t-test to assess the statistical significance of the difference in estimation errors between feature selection methods. Between RFSV and No Feature Selection, 12 out of the 25 subjects show statistical significance at the level alpha = 0.05 for both SBP and DBP. However, only 4 out of the 25 subjects show statistical significance when we compare RFSV and RFFI. This is consistent with the observation that feature selection can reduce the MAE, and RFSV performs just slightly better than RFFI in terms of MAE. We decide to use RFSV because of its lowest MAE and strong interpretability base on Game Theory.

**TABLE 5:** MAEs Using Different Feature Selection Methods

	No Feature Selection	PCFS	IGFS	RFFI	RFSV
SBP	5.79	5.64	5.71	5.36	5.34
DBP	3.95	3.87	3.92	3.83	3.80

### C. Personalized and Precise Recommendation

**FIGURE 5.**
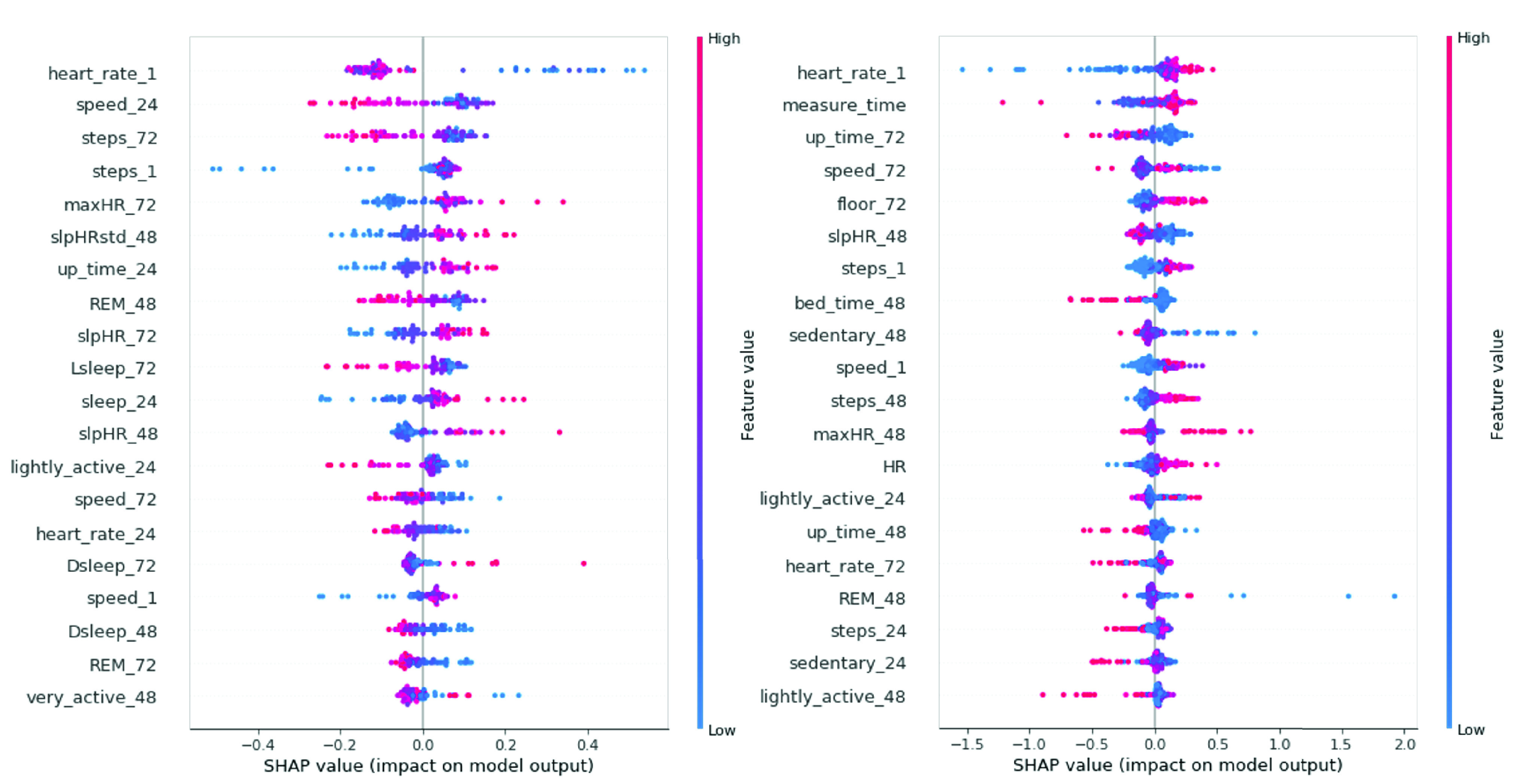
Left: SHAP values of features on SBP for subject 1. Right: SHAP values of features on SBP for subject 2.

**FIGURE 6.**
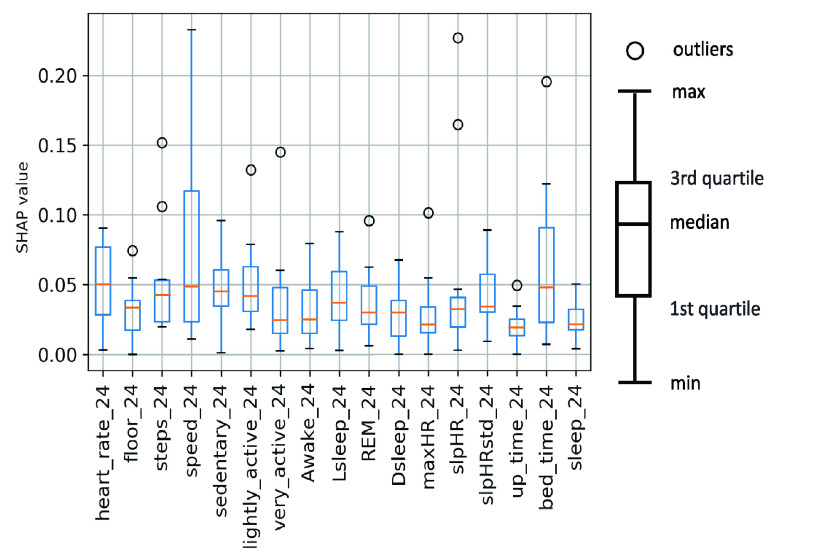
Box plot of SHAP values of lifestyle features (over the previous 24 hours).

In Fig. 5, we illustrate the contribution from each feature to an increase (or decrease) in SBP prediction for two subjects using SHAP [41]. Each dot represents the Shapley value for the feature listed on the Y-axis to the BP prediction of a sample. The placement on the X-axis represents the amount of positive/negative contribution to BP prediction. The color represents the actual value of the feature (red is high while blue is low). The feature list is sorted by contribution to the model from most to least. For example, from heart_rate_1 of subject 1 we can observe most blue dots (lower heart rate) are associated with higher BP prediction and most red dots (higher heart rate) are associated with lower BP. On the contrary, heart_rate_1 of subject 2 has the opposite relationship with his/her SBP. Moreover, the order of the top features from the two subjects is very different. The above observations confirm that different lifestyle factors may affect the BP of different individuals differently, with the top factors different for different individuals, and hence the motivation to provide personalized recommendations based on his/her data. With high granularity of lifestyle data collected from individuals and interpretation by Shapley values, patients and doctors can understand how lifestyle factors affect BP in a more precise and personalized fashion. In addition to using two subjects as examples to discuss the SHAP results above, we next expand the discussion to all subjects in this study. We first calculate the mean absolute value of SHAP values (which are the dots plotted in Fig. 5) of each feature. Based on the mean SHAP values of each subject, we provide a box plot of representative features over the previous 24 hours used in Sec. III-B to show the minimum, the maximum, the median, and the first and third quartiles of the SHAP values among all subjects, as shown in Fig. 6. We can observe that heart_rate24, speed_24, and bed_time_24 (the time when subjects go to sleep) have the highest median SHAP values while sleep_24 (total sleep duration) and up_time_24 (the time when subjects wake up) have the lowest median. Among the 17 features in Fig. 6, heart_rate24 has the highest SHAP values in 3 of 25 subjects; speed24 has the highest SHAP values in 7 of 25 subjects and bed_time_24 has the highest SHAP values in 5 of 25 subjects. For the other 10 subjects, their top features are not the three features with the highest median SHAP values shown. The above result validates our motivation to provide recommendations based on each subject’s SHAP values. However, the statistical analysis of SHAP values from all subjects may still provide valuable insights for designing population health management solutions.

**FIGURE 7.**
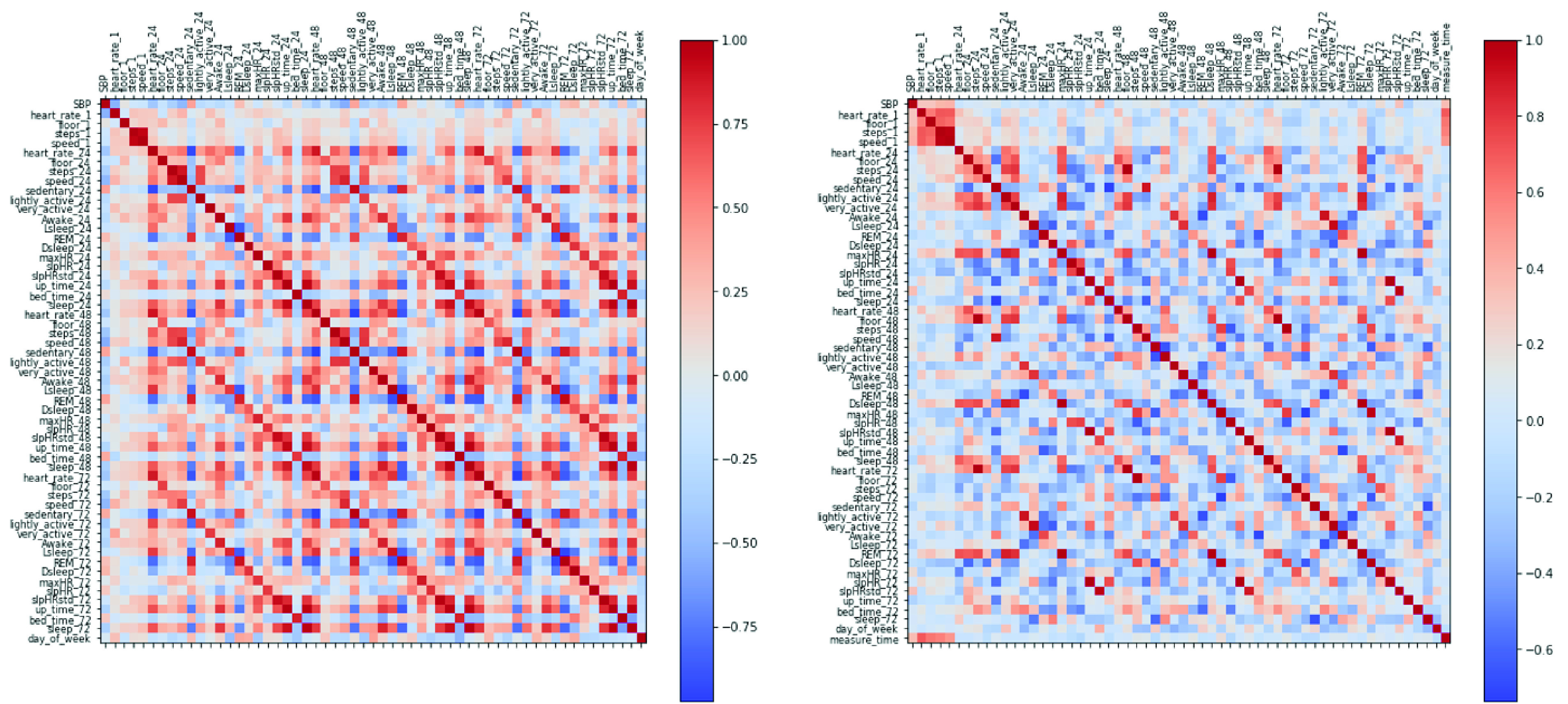
Left: Pearson Correlation Heatmap for subject 1. Right: Pearson Correlation Heatmap for subject 2.

To further validate the correlation between BP and the features, Fig. 7 displays a heatmap of the Pearson correlation between all features and SBP for the two subjects shown in Fig.5. For subject 1, the top three factors based on SHAP are heart_rate_1, speed_24 and steps_72, and all of them are negatively correlated to SBP. For subject 2, the top three factors based on SHAP are heart_rate_1 (positive correlation), measure_time (positive correlation) and up_time72 (negative correlation). We can observe the top features selected based on SHAP are largely consistent with the correlation heatmap, in terms of both direction and intensity.

**FIGURE 8.**
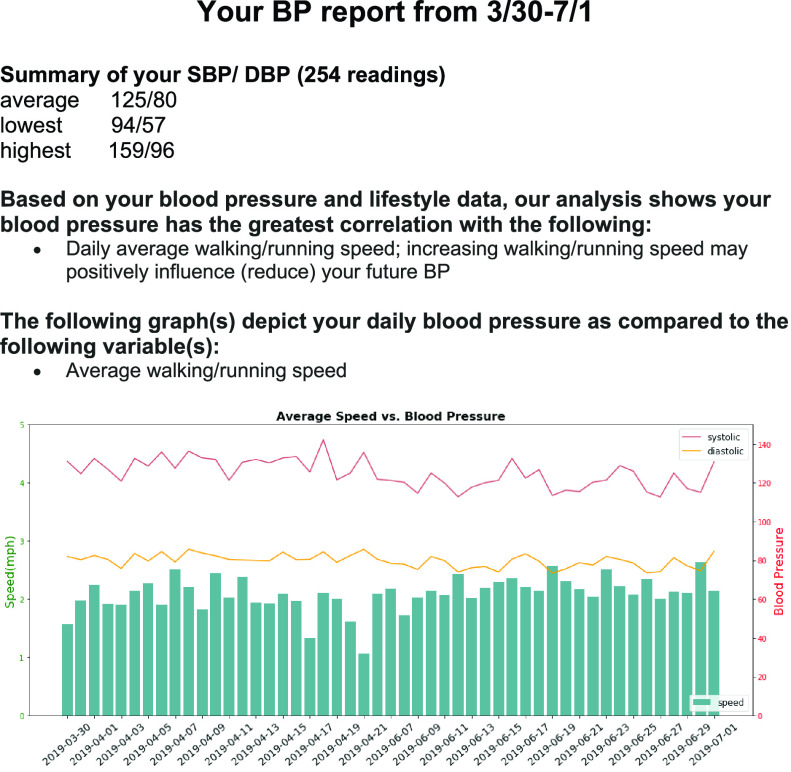
Example of personalized recommendation of subject 1.

To validate the effectiveness of the lifestyle recommendations suggested by the RFSV model, we randomly selected 6 of the 13 eligible subjects to form the experimental group which would receive personalized recommendations. That is, one month before the end of the study, we sent each subject in the experimental group an email consisting of 1) basic statistics of their BP during the study period, including the average, minimum and maximum blood pressure during the trial and 2) top lifestyle features for his/her BP prediction model based on Shapley value. The design of recommendation language for each feature is done in consultation with the physician collaborator in our research team. Lastly, we plot the figures which show the daily values of BPs and the corresponding top features to serve as subjects’ reference. An example of the recommendation for subject 1 is shown in Fig. 8. Although heart_rate_1 is the top feature for subject 1 (Fig. 5), it is not an actionable factor. Therefore, we recommend the next top feature, walking/running speed (speed_24), as the top factor. From Fig. 5, we can observe most red dots (higher speed) of speed_24 are associated with lower BP, so our personalized recommendations suggest the subject increase his walking/running speed. Following this email feedback, we collect BP data for a month for the experimental group and compare it with the control group.

The other 7 subjects are assigned to be in the control group which did not receive any feedback. As mentioned in Sec. IV-A, the other 12 subjects complete BP measurements across the study period, but they did not have enough lifestyle factor data collected from wearables. Those subjects are assigned to the control group since they had the same treatment (not receive feedback) as the 7 subjects in the control group and we only focus on their BP measurements.

In Table 6, we list the top features (recommendations) of the subjects in the experimental group and their BP statistics before and after receiving the recommendations. For the same type of feature in different time windows, we give the same recommendation without mentioning the time windows. For example, if user 1’s top feature is steps_24 and user 2’s top feature is steps_48, we will give both users the same recommendation as steps. The rationale is that features extracted based on different time windows may be useful to enhance predictive accuracy, but they are not intuitive for people to follow. From Table 6, we can observe that the top features can be very different for different subjects. For example, BP is mainly correlated to activity-related features for some subjects (1, 3, 4), sleep-related features for others (2, 5). To evaluate the change in BP, we calculate the mean and maximum of daily BP in the first week and the last week of the study since BP fluctuates with time and each single measurement may not reflect the actual BP of an individual. Additionally, we use longitudinal linear regression to calculate the linear slope of BP trend before and after receiving the recommendation to further understand their BP changes.

**TABLE 6:** Cohort Statistics and Summary of the BP Changes in the Experimental Group and the Control Group

	Before recommendation (SBP, DBP)				After recommendation (SBP, DBP)		
Subject ID	Mean BP	Max BP	slope	Recommendations based on top features	Mean BP	Max BP	slope
1	129, 81	142, 90	−0.12, −0.04	speed, steps	123, 80	139, 85	−0.27, −0.14
2	128, 82	137, 89	−0.04, −0.06	wake up time	120, 75	132, 79	−0.12, −0.21
3	117, 69	134, 79	0.05, 0.03	steps, floors	117, 68	122, 72	−0.08, 0.21
4	134, 83	157, 100	−0.04, −0.03	steps, speed	133, 80	142, 86	−0.27, −0.13
5	127, 82	152, 94	0.03, 0.01	steps, speed, sleep duration	121, 81	127, 85	−0.41, −0.32
6	122, 79	127, 91	0.04, 0.07	wake up time, floors	120, 78	126, 83	−0.08, −0.07

In Table 6, we show that the mean and maximum BP of most subjects in the experimental group improved (decreased) after the recommendation, except for subject 3 whose mean SBP remained the same. The average changes in mean BP for all subjects were −3.8 and −2.3 for SBP and DBP, respectively, and the average changes of maximum BP were −10.5 and −8.8 for SBP and DBP, respectively. For the slope of BP trend, we observe the BP trend turns from slightly increasing to decreasing for subjects 5 and 6 and from slightly decreasing to a steeper decreasing trend for subjects 1, 2 and 4. The exception is the DBP trend for subject 3. The change in BP varies significantly among the subjects where the change ranges from −10 to 0 points for mean BP and −25 to −1 points for maximum BP, from the first week to the last week of the study. One possible reason for such variation is that the stableness of BP and its correlation to lifestyle factors may differ among people. Finally, we discuss subject 3, whose BP remained mostly unchanged during the study. Although his BP records satisfied the initial subject screening criteria (SBP between 120–140 and DBP under 90), his measured BP was mostly recorded to be under 120/80 during the study. Therefore, lifestyle recommendations may have less effect on his BP which is already in a normal range.

Next, we compare the change of BP between the control group and the experimental group, as shown in Table 7. For consistency, we use the same method to derive the mean BP, max BP, and BP trend slopes for subjects in the control group. The average mean and max BPs of subjects in both groups decreased by the end of the study, suggesting a positive effect of awareness through only using the wearable device and measuring BP daily. However, the decreases in mean BPs (−3.8 and −2.3 for SBP and DBP) and max BPs (−10.5 and −8.8 for SBP and DBP) for the subjects in the experimental group are meaningfully greater than subjects in the control group, which are (−0.3, −0.9) and (−3.3, −2.5) for mean BPs and max BPs respectively. A two-sided Student’s t-test [49] is done to compare the reduction of mean BPs and max BPs for the two groups. The null hypothesis is that the mean BPs and the max BPs for the two groups have no difference. The p-values for mean BPs are 0.15 and 0.22 for SBP and DBP, and the p-values for max BPs are 0.07 and 0.05 for SBP and DBP respectively. The result does not reject the null hypothesis for significance level $\alpha =0.05$ except for max DBP. One possible reason for higher p-values is the impact of random error due to the smaller sample size, especially for the experimental group. Since the average changes cannot fully represent the individual effect, we also calculate the ratio of subjects in each group who improved (reduced) their mean and max BP. In the experimental group, 83% (5 of 6 subjects) and 100% (6 of 6 subjects) improved their mean SBP and DBP, respectively, compared to only 47% (9 of 19 subjects) and 53% (10 of 19 subjects) of the control group. Similarly, all subjects in the experimental group improved their max SBP and DBP, respectively, compared to only 63% and 58% of the subjects in the control group, respectively. Finally, in the last 30 days, the BP trend slope of subjects in the control group is relatively flat, while a decreasing trend is observed in the experimental group. In summary, subjects who received personalized recommendations about their lifestyle factors and BP were more likely to have demonstrated a decrease in their mean and max BP by the end of the study. Furthermore, the magnitude of this decrease in BP was greater in this experimental group compared to the control group.

**TABLE 7:** Recommendations and BP of Subjects in the Experimental Group Before and After their Personalized Recommendation

Measures	Experimental group (n = 6)		Control group (n = 19)	
Age	50.1+/- 15.0		52.9+/- 13.1	
Female ratio (%)	33%		37%	
	SBP	DBP	SBP	DBP
Initial BP (Mean +/- SD)	125.4+/-5.9	80.8+/-6.7	127.2+/-7.3	80.0+/-5.9
Mean BP change	−3.8	−2.3	−0.3	−0.9
Subjects with decreasing mean BP (%)	5 (83%)	6 (100%)	9 (47%)	10 (53%)
Max BP change	−10.5	−8.8	−3.3	−2.5
Subjects with decreasing max BP (%)	6 (100%)	6 (100%)	12 (63%)	11 (58%)
Trend slope in the last 30 days	−0.26	−0.13	−0.04	0.01

Limitations to this experiment include, by definition, the relatively small number of subjects who had complete BP and lifestyle data for analysis by study end (13 of the original 36 subjects enrolled), due to early participant drop-off and missing data. The lower ratio of eligible subjects demonstrates the universal challenge of keeping patients engaged in their health and the need to create more automated and convenient means of remote health monitoring. In addition, while this snapshot in time showed promising results, the lasting effect of any intervention is best demonstrated over longer study periods. In summary, while the results presented above are encouraging, future studies with a greater number of participants monitored over a longer duration are needed.

## V. Conclusion

In this paper, we investigate the personal effect of lifestyle factors on BP using data collected from wearables and home BP monitors, on 25 subjects in a clinical trial conducted in collaboration with UC San Diego Health and Altman Clinical and Translational Research Institute. Our proposed approach includes developing a personalized BP model for each individual using BP and lifestyle data for that individual, identify the most important lifestyle attributes that impact an individual’s BP trend and provide precise recommendations to improve the individual’s BP. Specifically, we propose a RFSV personalized model which we demonstrate can outperform other existing ML techniques in terms of prediction accuracy - by 10.1% and 6.2% in terms of MAE for SBP and DBP; 10.9% and 7.5% in terms of MAPE for SBP and DBP; 14.4% and 10.4% in terms of RMSE, for SBP and DBP respectively, and also achieving the highest R^2. We also propose a method based on Shapley value to identify the top features which affect the BP for each individual and provide personalized recommendations. Using a randomized control experiment, we show that significant improvement in BP can be achieved with personalized lifestyle recommendations. After receiving recommendations, the subjects in the experimental group decreased their BPs by 3.8 and 2.3 for systolic and diastolic BP, compared to a decrease of 0.3 and 0.9 for the subjects who did not receive recommendations.
